# Functional network of glycan-related molecules: Glyco-Net in Glycoconjugate Data Bank

**DOI:** 10.1186/1752-0509-4-91

**Published:** 2010-06-29

**Authors:** Ryo Hashimoto, Kazuko Hirose, Taku Sato, Nobuhiro Fukushima, Nobuaki Miura, Shin-Ichiro Nishimura

**Affiliations:** 1 Division of Advanced Chemical Biology, Graduate School of Life Science, Frontier Research Center for Post-Genomic Science and Technology, Hokkaido University, Sapporo 001-0021, Japan; 2 Hokkaido STS, Inc., 5-1-16 Hongo, Bunkyo-ku, Tokyo 113-0033, Japan; 3 Sun Microsystems Laboratory for Computational Molecular Life Science, Graduate School of Life Science, Hokkaido University, Sapporo, 001-0021, Japan; 4 Drug-Seeds Discovery Research Laboratory, Hokkaido Center, National Institute of Advanced Industrial Science and Technology (AIST), Sapporo 062-8517, Japan

## Abstract

**Background:**

Glycans are involved in a wide range of biological process, and they play an essential role in functions such as cell differentiation, cell adhesion, pathogen-host recognition, toxin-receptor interactions, signal transduction, cancer metastasis, and immune responses. Elucidating pathways related to post-translational modifications (PTMs) such as glycosylation are of growing importance in post-genome science and technology. Graphical networks describing the relationships among glycan-related molecules, including genes, proteins, lipids and various biological events are considered extremely valuable and convenient tools for the systematic investigation of PTMs. However, there is no database which dynamically draws functional networks related to glycans.

**Description:**

We have created a database called Glyco-Net http://www.glycoconjugate.jp/functions/, with many binary relationships among glycan-related molecules. Using search results, we can dynamically draw figures of the functional relationships among these components with nodes and arrows. A certain molecule or event corresponds to a node in the network figures, and the relationship between the molecule and the event are indicated by arrows. Since all components are treated equally, an arrow is also a node.

**Conclusions:**

In this paper, we describe our new database, Glyco-Net, which is the first database to dynamically show networks of the functional profiles of glycan related molecules. The graphical networks will assist in the understanding of the role of the PTMs. In addition, since various kinds of bio-objects such as genes, proteins, and inhibitors are equally treated in Glyco-Net, we can obtain a large amount of information on the PTMs.

## Background

Glycans are involved in a wide range of biological process, and they play an essential role in functions such as cell-cell interaction, pathogen-host recognition, toxin-receptor interaction, signal transduction.[1-5] One of their roles are modulating the functions of many proteins and lipids through post-translational modifications (PTMs).[6] Glycomics is the study of the structural and functional aspects of various glycoconjugates, such as glycoproteins, glycolipids, and proteoglycans produced during PTMs in cells and organisms. The field of glycomics has lagged behind that of genomics and proteomics, mainly because of the inherent difficulties in the analysis of glycan structure and function.[7] However, glycomics is now an emerging field due to exceptional progress in the development of modern experimental techniques and equipment including mass spectrometry (MS), high-performance liquid chromatography (HPLC), nuclear magnetic resonance (NMR) and knockout mice.[8-15] It is expected that a large quantity of information concerning glycan structure and function will be accumulated. Bioinformatics of glycans, which used to suffer from a lack of data in early studies, is now becoming a practical field in the biological sciences related to PTMs. Therefore, the construction of a new class glycan database indicating the relationship between structures and their functions and the development of related tools is strongly required from biological, pharmaceutical and medical fields.

There are several groups energetically developing both public and commercial glycan databases. For instance, some of the public databases are KEGG [16-18], SWEET-DB [19] in the GLYCOSCIENCES.de [20], the United States Consortium for Functional Glycomics (CFG) [21], and GlycoSuiteDB in The Expert Protein Analysis System (ExPASy) Proteomics Server [22]. GlycoMinds http://www.glycominds.com is known as the commercial database. The Complex Carbohydrate Structure Database (CCSD) [23,24] is the first database of glycan structures. The CCSD was developed in the 1980s and 1990s by the CarbBank Project and was discontinued in 1999 due to the lack of funding. The data of the CCSD are currently included in the public glycan databases as mentioned above. Although the web service of GLYCOSCIENCES.de is currently not available, they are trying to organize the new base for their database. The Carbohydrate-Active Enzyme (CAZy) database is known as a database of enzymes relating to glycans, such as glycosyltransferases and lectins [25]. All of these databases with the exception of CAZy are focused on glycan structures. The SWEET-DB mainly develops the tools with which to treat the glycan structures and geometry [26-29]. The CFG is constructing carbohydrate chips to investigate the interaction between carbohydrates and proteins for therapy, and databases for functional glycomics, such as an annotated database of mass spectrometry. The KEGG GLYCAN database also has over 10,000 glycan structures; in addition, a manually drawn graphical pathway for various bio-molecules is included in KEGG PATHWAY. The Expert Protein Analysis System (ExPASy) http://www.expasy.org/ which includes a protein sequence database also holds many graphical figures of biochemical pathways. Krambeck and Betenbaugh [30] and Liu et al. [31] have developed a system which dynamically constructs a structural network regarding *N*- and *O*-linked glycans, respectively.

Recently, emerging analytical techniques enabled us to obtain a great deal of information about the relationships, not only between the glycan structures and functions, but also among glycans, phenotypes of diseases and expression of glycan-related genes. In this situation, graphical networks describing the relationships among glycan-related molecules, including genes, proteins, lipids and biological events are considered to become potential tools for accelerating the integrated study of PTMs. Although the KEGG PATHWAY and Biochemical Pathways in ExPASy http://us.expasy.org/cgi-bin/show_thumbnails.pl have graphical network figures, these are all manually selected and organized. Since glycomics and glycoproteomics data are expected to increase substantially, it is clear that the network figures generated from the glycan structures should be drawn based on the available updated data in order to give the most current overview of glycan functions.

We have endeavoured to dynamically draw figures of functional networks among glycans, genes, inhibitors, lipids, glycosphingolipids, various biological events, diseases and carbohydrate-binding proteins such as glycosyltransferases and lectins (hereafter, these are denoted as "bio-objects") for several years. Dynamic generation of the network figures within bio-objects is more progressive than networks of biosynthesis with static pictures such as KEGG PATHWAY and ExPASy. Glyco-Net was constructed as a part of the Glycoconjugate Data Bank (GDB) http://www.glycoconjugate.jp/. Each bio-object in Glyco-Net is linked to the other databases to obtain more detailed information, since Glyco-Net has been focusing on the collection of the functional relationships among bio-objects from research articles. In this paper, we describe the concept and status of Glyco-Net.

## Construction and content

### Basic concepts

**Figure 1:**
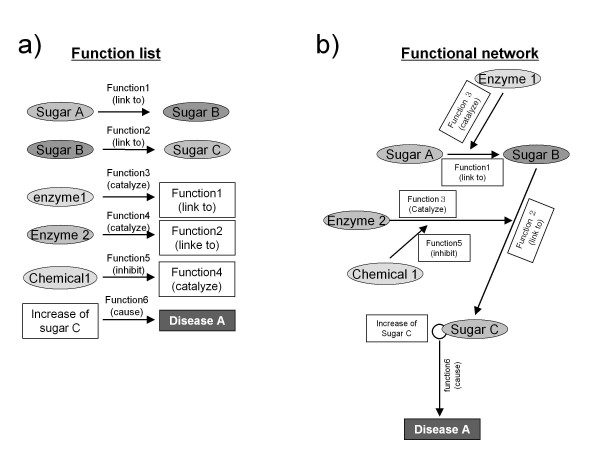
**Concept of Glyco-Net**. a) Function list. Two bio-objects (or functions) are connected with an arrow. The function on the third row of Figure 1a shows "enzyme1 catalyzes (Function3) Function1". This is an example of a function behaving as a node. b) Functional network made from a), superimposing the same bio-objects and functions.

Glyco-Net dynamically draws functional networks in a variety of bio-objects relating to known glycans. Binary relations among glycan-related information are accumulated, as shown on the left side of Figure 1. Two bio-objects are connected by a "verb" which expresses the function of a bio-object, for example "Sugar A links to Sugar B" in Figure 1a. A function "A link to" means alpha linkage of formed glycoside bond after sugar transformation. A bio-object is described as a node, and a "verb" is expressed as an arrow in the functional network. We use over 100 verbs in Glyco-Net which are listed in Additional file 1. In order to make a network figure, the same bio-objects are superimposed at the node, which is shown on the right hand side of Figure 1b. The binary relationships for bio-objects have been curated manually. We are now constructing a kind of ontology in the wide field of glycan-related research in order to automatically gather the above binary relationships from the web articles such as PubMed http://www.pubmed.org, and this will be discussed further in our future articles. This ontology will be a powerful tool in the near future even though we should verify the data by sight.

The functional networks are dynamically created by using the stacked binary relationships. Since all bio-objects (nodes) and functions (arrows) are treated equally in Glyco-Net, an arrow behaves as a node in some cases. This feature enables us to draw flexible networks. Glyco-Net also holds a linkage which is displayed in the object tables to obtain the detailed information of the bio-objects from the other biological databases, though the linkages are not available from the network figures. Therefore the development of clickable linkage on the network figures would lead to the further examination. Following linkages are listed in Table 1: the genes entries hold the linkage to search the gene in GenBank [32], sugar entries have the linkages to KEGG GLYCAN, the enzyme entries have the linkage to the enzyme database ENZYME in ExPASy, and the disease entries have linkage to a disease database such as Online Mendelian Inheritance in Man (OMIM) and literature databases such as PubMed.

**Table 1:** List of linkages from Glyco-Net

Bio-object	Data base	URL and comments
SUGAR	KEGG GLYCAN	http://www.genome.jp/dbget-bin/www_bfind?glycan
		Only whole sugar structures, we can not define the linkage to partial structures.
PROTEIN	ExPASy	http://www.expasy.org/enzyme/
	Proteomics Server	This linkage is defined through the EC number of protein.
GENE	GenBank	http://www.ncbi.nlm.nih.gov/Genbank/index.html
DISEASE	OMIM	http://www.ncbi.nlm.nih.gov/entrez/query.fcgi?db=OMIM
CELL	CELL BANK	http://www.brc.riken.jp/lab/cell/
ARTICLE	PubMed	http://www.ncbi.nlm.nih.gov/entrez/query.fcgi?db=PubMed

Glyco-Net is expected to be used as an interface between the various biological databases and the functional network of glycan-related bio-objects. The current notation of carbohydrate structures is ad hoc. There are various structural databases, such as KEGG GLYCAN, GLYCOSCIENCES.de, and CFG. Thus, it is only necessary to give the linkage from our glycan data to enter these databases. At the moment, Glyco-Net holds limited linkage to carbohydrate structure databases. We will modify the nomenclature of the carbohydrate structures with a more standard one, such as GLYDE [33], to make it accessible in other databases with the carbohydrate structure as a key.

Implementation of our database was carried out with a MySQL database system and a Linux environment. The interface web page was written in JavaServer Pages (JSP). The search engine and the drawing method were written in Java Programming language.

### Data curation

Glyco-Net aims to collect binary relations that could be extracted by going through the scientific articles such as research papers, i.e. evidence of functions by specific assays. These data were manually curated from the "*Handbook of Glycosyltransferases and Related Genes*." [34] Functions with different experimental conditions in the assay are all recognized as different functions and existed in the network figure at the same time. It is necessary to classify the functions with ontology according to the experimental conditions and/or the environment where the bio-objects are in so that the quantitative discussion can be carried out.

### Data structure and statistics

Glyco-Net consists of four categories of data which are shown in Table 2. The first category is the "function", which describes the relationships between biological objects. The second one is the "object", which describes the detailed information of biological objects such as genes, proteins, lipids, glycans, and diseases. The third one is the "assay", which provides information on assays from which the functions are suggested. In addition, the references are found in the "article" category. These categories of data are divided into several tables, for example, the "object" is divided into seven tables called "sugar", "protein complex", "protein", "gene", "lipid", "disease" and "event". Relationships between articles and other tables are described in the "reference" table. Detailed data structures are provided in the Additional file 1.

**Table 2:** Annotation and data in Glyco-Net

Attribute of data	Description	Number of data
FUNCTION	Relationships between two objects.	2,302
BIO-OBJECT	Description of the object which constructs the functions, such as carbohydrates, related genes, glycosyltransferases, lipids, glycolipids, diseases, biological events, etc.	3,724
ASSAY	Experimental information which elucidates the functions.	1,201
ARTICLE	Reference data.	1,332

Currently, Glyco-Net has 3,724 objects (1,149 objects for glycosyltransferases, 2,480 objects for genes, and 95 pieces of data concerning diseases caused by or related to carbohydrate abnormalities), 2,302 pieces of function data, and 1,201 pieces of data concerning the assay that verifies the functions of the glycoconjugates. Records (1,332) are also contained in the "article" category. Data which referenced from any articles that published after Reference 34 will be updated in the future. Furthermore, we have been developing ontology regarding Glyco-Net.

## Utility and Discussion

### Access to Glyco-Net

The main page of the Glycoconjugate Data Bank http://www.glycoconjugate.jp provides three links to databases, including 1) "Resources", which is a database of carbohydrate-related compounds, 2) "Structure" [35], which is a 3D structure database of glycans extracted from the Protein Data Bank and 3) "Glyco-Net", which shows the functional network of carbohydrate-related molecules. We can browse several function lists and network figures by clicking the bio-object type or typing the keyword to see the details of the functions.

### Simple examples of searching Glyco-Net

**Figure 2:**
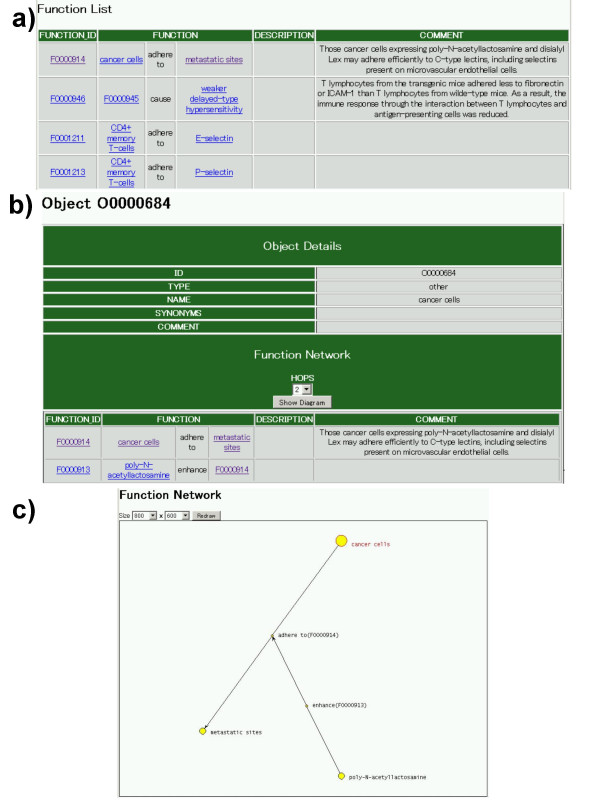
**A search result in Glyco-Net**. a) Search results with the word "adhere". b) A detail of bio-object cancer cell (O0000684). This table has ID, TYPE, NAME, SYNONYMS, and COMMENT fields. The bottom of this table shows the function list corresponding to a certain HOPS. c) A dynamic network figure for bio-object O0000684 within HOPS of 2.

Figure 2 shows a simple example of the search results from Glyco-Net. Figure 2a shows the result of keyword search "adhere" and several functions were found. The function list includes the function ID, the function itself, a detailed description of the function, and the comments in the function tables. Figure 2b shows the details of an object "cancer cell". In the table, the object ID, the object type, the object name, the synonyms, and the comments of the object are shown. The list of functions with the objects is shown in the part of the table. The "HOPS" number refers to the number of nodes from the object which consists of functions or objects in the table linked to the selected object of "cancer cells". In this sample search, the HOPS was set to 2. Currently, HOPS was limited to five in Glyco-Net due to our technical problem. The limitation of the HOPS number could cause ambiguous results by partly drawing network figures. In order to obtain fully accurate network figures, we are developing a novel drawing method and will update the drawing routine in the near future. By clicking the "Show Diagram" button, a figure of the functional network is shown as Figure 2c. Topology in the figure might vary as redrawing. In addition, the resolution of the figure can be changed by selecting the size of the figure. The default size of the figure is 1024 × 768 dots. Figure 2c shows a simple example that a function "Cancer cells adhere to metastatic sites." (Function ID is F0000914) is enhanced by poly-*N*-acetyllactosamine (Function ID is F0000913). Furthermore, since all bio-objects are treated equally, arrows have a node that is the same as other bio-objects such as "cancer cell". This is a characteristic feature of Glyco-Net. According to the value of the HOPS number, the networks would grow substantially. However, the network figures are quite complicated for a large HOPS.

**Figure 3:**
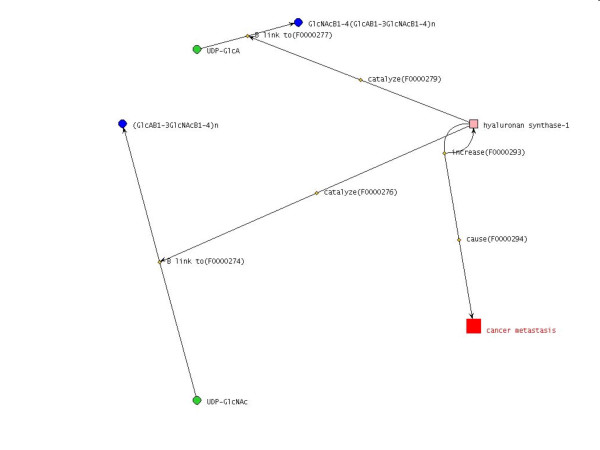
**Function network figure of functions of hyaluronan synthase-1**. This network figure shows that 1) an increase of hyaluronan synthase-1 causes cancer metastasis, 2) hyaluronan synthase-1 catalyses a Glcuronic acid transfer reaction, and 3) hyaluronan synthase-1 also catalyzes a GlcNActransfer reaction (see details in the text).

Figure 3 shows a slightly more complex network than Figure 2c. The "cause" is the function that increases in hyaluronan synthase-1 causes cancer metastasis. Any function which relates to the object itself is expressed as a round arrow returning to the object. From the rest of the network figure we can discern that hyaluronan synthase-1 catalyzes two glycosylation reactions, including the formation of glycoside linkages between glucuronic acids (GlcA) and GlcNAcβ1-4(GlcAβ1-3GlcNAcβ1-4)_*n *_and between *N*-acetylglucosamine (GlcNAc) and (GlcAβ1-3GlcNAcβ1-4)_*n*_. This is consistent with the actual function of the hyaluronan synthase-1 that synthesizes hyaluronic acids which are comprised of repeats of the GlcA-GlcNAc disaccharide unit.

### Conclusions

In this paper, we describe our new database, Glyco-Net, which shows graphical networks of glycan-related bio-objects such as genes, proteins, glycoproteins, lipids, glycolipids, and glycans. Each bio-object can easily be linked to the available databases such as GenBank, ExPASy, KEGG GLYCAN, GLYCOSCIENCES.de, CFG, and PubMed, though the linkage is limited from the bio-object tables at the present time. Dynamic generation of the functional network figures among bio-objects is expected to have great advantages compared with KEGG PATHWAY and ExPASy which hold static figures for biosynthesis. Since various kinds of bio-objects such as genes, proteins and inhibitors are equally treated in Glyco-Net, a large amount of information on the PTMs can be obtained. Although these characteristics are the novel implementation in the existing glycan databases, figures made by Glyco-Net are still complicated to adapt to a larger HOPS at this stage. In addition, the quantity of total data in Glyco-Net still remains a small. Therefore, we are now constructing ontology for partly automatic curation from web articles. An automatic curation with ontology will become a quite powerful tool, even though collected data should be verified carefully by scientists. We will also develop a routine to clearly draw the functional network figures. Furthermore, the nomenclature of the glycan structure should be standardized in order to search the glycans in other structure-based carbohydrate databases without uncertainty. Use of GLYDE notation is found to be quite feasible, since only our database indicates the relationships among biological objects relating to glycans. As a result, the details of the objects have to be found in other databases, and we will have to increase the linkages from our objects to other databases. Thus, the establishment of the collaboration with researchers in bioinformatics and other biosciences to improve this new type of database is the significant asset for the further development of Glyc-Net.

## Availability and requirements

URL of Glyco-Net is http://www.glycoconjugate.jp/functions/.

## Authors' contributions

RH designed and constructed the database. NM drafted the manuscript. KH, TS, and NF participated in data curation. SN supervised the whole project. All authors read and approved the final manuscript.

## Supplementary Material

Additional File 1 **Supplemental materials data structure and list of verbs in Glyco-Net are given as the supplemental materials**. Click here for file
