# Supplementary material for: Functional network of glycan-related molecules: Glyco-Net in Glycoconjugate Data Bank
Source: BMC Syst Biol. 2010 Jun 29;4:91. doi: 10.1186/1752-0509-4-91 (PMC2907334; doi:10.1186/1752-0509-4-91)
Supplement: Additional File 1 — Supplemental materials data structure and list of verbs in Glyco-Net are given as the supplemental materials. [file 1752-0509-4-91-S1.DOC]

# Functional network of glycan-related molecules: Glyco-Net in Glycoconjugate Data Bank

Supplemental Materials

### Ryo Hashimoto^1,2^, Kazuko Hirose^1,2^, Taku Sato^2^, Nobuhiro Fukushima^2^, Nobuaki Miura^3§^ and Shin-Ichiro Nishimura^1,4§^

^1^ Division of Advanced Chemical Biology, Graduate School of Advanced Life Science,Frontier Research Center for Post-Genomic Science and Technology, Hokkaido University, Sapporo 001-0021, Japan

^2^ Hokkaido STS, Inc., 5-1-16 Hongo, Bunkyo-ku, Tokyo 113-0033, Japan

^3^ Sun Microsystems Laboratory for Computational Molecular Life Science, Graduate School of Advanced Life Science, Hokkaido University, Sapporo, 001-0021, Japan

^3^ Drug-Seeds Discovery Research Laboratory, Hokkaido Center, National Institute of Advanced Industrial Science and Technology (AIST), Sapporo 062-8517, Japan

^§^Corresponding author

Email addresses:

RH: [rhashimo@glyco.sci.hokudai.ac.jp](mailto:rhashimo@glyco.sci.hokudai.ac.jp)

KH: [hirose@glyco.sci.hokudai.ac.jp](mailto:hirose@glyco.sci.hokudai.ac.jp)

TS: [m801m802@me.com](mailto:m801m802@me.com)

FN: [fukushima@st-systems.co.jp](mailto:fukushima@st-systems.co.jp)

NM: [muller@glyco.sci.hokudai.ac.jp](mailto:muller@glyco.sci.hokudai.ac.jp)

SN: [shin@glyco.sci.hokudai.ac.jp](mailto:shin@glyco.sci.hokudai.ac.jp)

**S1 Data table structure**

| **◎OBJECT** |  |
| --- | --- |
| OBJECT_ID | an arbitrary ID for OBJECT |
| TYPE | any of the following: protein, protein complex, lipid, sugar, nucleotide, sugar nucleotide, event, proteoglycan, glycosaminoglycan, disease, gene, peptide, other |
| NAME | name of OBJECT |
| COMMENT | special mention for OBJECT |
|  |  |
| **◎SYNONYMS** |  |
| NAME | name of OBJECT that has synonyms |
| SYNONYMS | their synonyms |
|  |  |
| **◎SUGAR** |  |
| OBJECT_ID | OBJECT_ID for sugar(OBJECT TYPE) |
| CLASS | any of the following: monosaccharide, oligosaccharide, N-glycan, O-glycanから選択 |
| KEGG | the KEGG ID that links to KEGG Glycan(http://www.genome.jp/dbget-bin/www_bfind?glycan) |
|  |  |
| **◎FUNCTION** |  |
| FUNCTION_ID | an arbitrary ID for FUNCTION |
| A | OBJECT_ID |
| RELATION | a verb which expresses a relationship between A and B |
| B | OBJECT_ID |
| DESCRIPTION | descriptions of FUNCTION |
| LOCATION | special location for FUNCTION |
| COMMENT | special mention for FUNCTION |
|  |  |
| **◎FUNCTION_ASSAY** |  |
| FUNCTION_ID | the corresponding FUNCTION_ID |
| ASSAY_ID | the corresponding ASSAY_ID |
|  |  |
| **◎PROTEIN** |  |
| OBJECT_ID | OBJECT_ID for protein(OBJECT TYPE) |
| FUNCTIONAL_CLASS | the EC number links to ExPASy( http://www.expasy.org/enzyme/) |
| STRUCTURAL_CLASS | example) type1 membrane protein |
| FUNCTIONAL_GROUP | example) N-glycan processing enzyme |
| COMMENT | special mention for PROTEIN |
|  |  |
| **◎PROTEIN_COMPLEX** |  |
| PROTEIN_COMPLEX_OBJECT_ID | OBJECT_ID for protein complex(OBJECT TYPE) |
| PROTEIN_OBJECT_ID | the corresponding OBJECT_ID for protein |
|  |  |
| **◎GENE** |  |
| GENE_ID | OBJECT_ID for gene(OBJECT TYPE) |
| GENENAME | name of GENE |
| OBJECT_ID | the corresponding OBJECT_ID for protein |
| ORGANISM | a name of organism that the gene comes from |
| GB_PROTEIN | GENBANK_ID(protein) links to Genbank(http://www.ncbi.nlm.nih.gov/Genbank/index.html) |
| GB_MRNA | GENBANK_ID(mRNA) links to Genbank(http://www.ncbi.nlm.nih.gov/Genbank/index.html) |
| GB_GENOMIC | GENBANK_ID(genome) links to Genbank(http://www.ncbi.nlm.nih.gov/Genbank/index.html) |
|  |  |
| **◎LIPID** |  |
| OBJECT_ID | OBJECT_ID for lipid(OBJECT TYPE) |
| CLASS | any of the following: Simple lipid, Glycerophospholipid, Sphingophospholipid, Glycoglycerolipid, Sphingoglycolipid |
|  |  |
| **◎DISEASE** |  |
| OBJECT_ID | OBJECT_ID for disease(OBJECT TYPE) |
| OMIM_number | OMIM_number links to OMIM(http://www.ncbi.nlm.nih.gov/entrez/query.fcgi?db=OMIM) |
|  |  |
| **◎EVENT** |  |
| OBJECT_ID | OBJECT_ID for event(OBJECT TYPE) |
| COMMENT | special mention for EVENT |
|  |  |
| **◎ASSAY** |  |
| ASSAY_ID | an arbitrary ID for ASSAY |
| ASSAY_TYPE | any of the following: CHEMICAL_ASSAY, CELL_ASSAY, INVIVO_ASSAY |
| COMMENT | special mention for ASSAY |
|  |  |
| **◎ACTIVITY** |  |
| OBJECT_ID | OBJECT_ID for protein(OBJECT TYPE) that shows Km value |
| DONER_SUBSTRATE | a name of doner substrate: examples) UDP-Man,　GDP-Fuc etc |
| KM_DONER | Km value of a doner substrate |
| ACCEPTER_SUBSTRATE | a name of accepter substrate |
| KM_ACCEPTER | Km value of an accepter substrate |
| COMMENT | special mention for ACTIVITY |
| ARTICLE_ID | the corresponding ARTICLE_ID |
|  |  |
| **◎INVIVO_ACTIVITY** |  |
| OBJECT_ID | OBJECT_ID for protein(OBJECT_TYPE) that shows activity in vivo |
| ORGANISM | a name of organism |
| TISSUE | a name of tissue: example) liver ,skin etc |
| LEVEL | examples) high, low, normal, weak, NO etc |
| TERM | examples) child, fetal etc |
| COMMENT | special mention for INVIVO_ACTIVITY |
| ASSAY_ID | the corresponding ASSAY_ID |
|  |  |
| **◎CELL_ASSAY** |  |
| ASSAY_ID | ASSAY_ID for CELL_ASSAY(ASSAY_TYPE) |
| CELL_NAME | a name of cell line |
| CELL_ID | cell ID links to CELL_BANK（http://www.brc.riken.jp/lab/cell/) |
| ORGANISM | a name of organism that the cell line comes from |
| TISSUE | a name of tissue: examples) liver ,skin etc |
| GENOTYPE | examples) GnT-III knockout, B3-GalT-3 over expression etc |
| COMMENT | special mention for CELL_ASSAY |
|  |  |
| **◎CHEMICAL_ASSAY** |  |
| ASSAY_ID | ASSAY_ID for CHEMICAL_ASSAY(ASSAY_TYPE) |
| CHEMICAL_CONDITION | descriptions of conditions for CHEMICAL_ASSAY |
| COMMENT | special mention for CHEMICAL_ASSAY |
|  |  |
| **◎INVIVO_ASSAY** |  |
| ASSAY_ID | ASSAY_ID for INVIVO_ASSAY(ASSAY_TYPE) |
| ORGANISM | a name of organism |
| GENOTYPE | examples) GnT-III knockout, B3-GalT-3 over expression etc |
|  |  |
| **◎EXPRESSION** |  |
| OBJECT_ID | OBJECT_ID for protein(OBJECT_TYPE) or gene(OBJECT_TYPE) |
| ORGANISM | a name of organism |
| TISSUE | a name of tissue |
| LEVEL | expression level: examples) high, low, normal, weak, NO etc |
| TERM | expression term: examples) child, fetal etc |
| COMMENT | special mention for EXPRESSION |
| ASSAY_ID | the corresponding ASSAY_ID |
|  |  |
| **◎ARTICLE** |  |
| ARTICLE_ID | an arbitrary ID for ARTICLE |
| PM_ID | PubMed_ID links to PubMed(http://www.ncbi.nlm.nih.gov/entrez/query.fcgi?db=PubMed) |
| YEAR | a publication year |
| TITLE | a title of journal |
| JOURNAL | an abbreviation of journal |
| VOLUME | a volume of journal |
| AUTHOR | a name of first author |
|  |  |
| **◎REFERENCE** |  |
| ARTICLE_ID | the corresponding ARTICLE_ID |
| ASSAY_ID | the corresponding ASSAY_ID |
|  |  |
|  |  |

**S2 List of verbs in Glyco-Net**

| a simple verb that shows a relationship between A and B | | |  |  |
| --- | --- | --- | --- | --- |
|  | | |  |  |
| ***Category I*** | | | | |
| **evaluation-verbs** | determine | enable |  |  |
| ***Category II*** | | | | |
| **development-verbs** | become | develop | express | overexpress |
| **induction and production-verbs** | | catalyze | cause | induce |
| produce | stimulate | lead to |  |  |
| **increase and promotion-verbs** | | activate | dramatic increase | accelerate |
| attack | enhance | grow | increase | potentiate |
| elevate | promote | reactivate | transactivate | upregulate |
| **decrease and inhibition-verbs** | | abolish | abrogate | block |
| inhibit | competitive inhibit | | noncompetitive inhibit | |
| strongly inhibit | reduce | resist | is resistant to | impair |
| inactivate | degrade | slow | decrease | deactivate |
| downregulate | lose | suppress |  |  |
| **alteration-verbs** | recover | abnormalize | alter | change |
| trim | dehydrogenate | deoxygenate | deplete | galactoylate |
| worsen | hydroxylate | is deacetylated | isomerize | knockout |
| shift to | metastasize | modify | mutate | phosphorylate |
| transcript | transfect | transport |  |  |
| ***Category III*** | | | | |
| **relationship-verbs** | correlate with | involve | involve in | relate to |
| is associated with |  |  |  |  |
| **property-verbs** | accumulate | is essential for | direct | exist in |
| require | have | is a ortholog of | is a | consist of |
| separate | is in | is on | lack | locate in |
| survive | need | organize | rate-limit | release |
| **function-verbs** | a link to | B link to | link to | adhere to |
| accept | bind to | compensate for | control | correct |
| stabilize | donate | don't accept | don't recognize | form |
| recognize | govern | regulate | target |  |
| ***Category IV*** | | | | |
| **method-verbs** | add to | crystallize | (total 110 verbs) | |
